# Global Decline in Suitable Habitat for *Angiostrongylus* ( = *Parastrongylus*) *cantonensis*: The Role of Climate Change

**DOI:** 10.1371/journal.pone.0103831

**Published:** 2014-08-14

**Authors:** Emily M. York, Christopher J. Butler, Wayne D. Lord

**Affiliations:** 1 W. Roger Webb Forensic Science Institute, University of Central Oklahoma, Edmond, Oklahoma, United States of America; 2 Department of Biology, University of Central Oklahoma, Edmond, Oklahoma, United States of America; Institut de Biologia Evolutiva - Universitat Pompeu Fabra, Spain

## Abstract

Climate change is implicated in the alteration of the ranges of species worldwide. Such shifts in species distributions may introduce parasites/pathogens, hosts, and vectors associated with disease to new areas. The parasite *Angiostrongylus* ( = *Parastrongylus*) *cantonensis* is an invasive species that causes eosinophilic meningitis in humans and neurological abnormalities in domestic/wild animals. Although native to southeastern Asia, *A. cantonensis* has now been reported from more than 30 countries worldwide. Given the health risks, it is important to describe areas with potentially favorable climate for the establishment of *A. cantonensis*, as well as areas where this pathogen might become established in the future. We used the program Maxent to develop an ecological niche model for *A. cantonensis* based on 86 localities obtained from published literature. We then modeled areas of potential *A. cantonensis* distribution as well as areas projected to have suitable climatic conditions under four Representative Concentration Pathways (RCP) scenarios by the 2050s and the 2070s. The best model contained three bioclimatic variables: mean diurnal temperature range, minimum temperature of coldest month and precipitation of warmest quarter. Potentially suitable habitat for *A. cantonensis* was located worldwide in tropical and subtropical regions. Under all climate change RCP scenarios, the center of the projected distribution shifted away from the equator at a rate of 68–152 km per decade. However, the extent of areas with highly suitable habitat (>50%) declined by 10.66–15.66% by the 2050s and 13.11–16.11% by the 2070s. These results conflict with previous studies, which have generally found that the prevalence of tropical pathogens will increase during the 21st century. Moreover, it is likely that *A. cantonensis* will continue to expand its current range in the near future due to introductions and host expansion, whereas climate change will reduce the total geographic area of most suitable climatic conditions during the coming decades.

## Introduction

Changes in the distribution and phenology of many organisms were observed as the earth warmed by 0.6±0.2°C during the 20th century [1]–[4]. Since 1945, warming of the earth has been greater than any other time during the past 1,000 years [5]. Changing climate is predicted to drive 11% to 58% of vertebrate, invertebrate, and plant species to extinction by 2050 [6], and is also expected to promote expansion and/or geographic shift of tropical diseases into temperate areas [7]. Consequently, there is an urgent need to examine and model how climate change might alter infectious disease emergence within human, domestic, and wild animal populations worldwide [8].

Ecological niche modeling (ENM) predicts the fundamental and realized niche of species by relating point occurrence data of species to environmental factors [9], [10]. These models are useful in predicting the geographic range in which a species might be found, but are limited by the exclusion of detailed environmental characteristics (e.g. biotic interactions, heterogeneous landscapes). Maximum Entropy (Maxent) modeling uses environmental conditions and species presence only data to accurately estimate the distribution of a species [11]. By predicting the entire geographic range in which a species might occur, the fundamental niche of an organism is not limited by its realized niche. This approach can assess the relative importance of specific environmental factors to a species distribution, locate areas of current suitable habitat, and project changes in its distribution over time [11].

Epidemiology uses a multifaceted approach to monitor, predict and prevent disease outbreaks. ENM is a valuable epidemiological tool because it determines the functional geographic responses of parasites and pathogens to climate change, both proximate and future. Recent studies have incorporated ENM to assess the potential impacts of climate change on infectious diseases vectors, reservoirs and/or pathogens (e.g., leishmaniasis, monkeypox, Chagas’ disease, malaria and blastomycosis) [12]–[18].

*Angiostrongylus ( = Parastrongylus) cantonensis* is a parasitic nematode and a cause of the reemerging zoonotic disease, human eosinophilic meningitis, as well as neurological abnormalities in wildlife and domestic animals [19], [20]. Definitive and intermediate hosts for the parasite include rats and mollusks, respectively [21], [22]. Humans and other mammals are incidental hosts that become infected upon consumption of the third-stage larvae. Infection primarily occurs by consuming raw or undercooked mollusks or other infected paratenic hosts (e.g., freshwater prawns, frogs, monitor lizards) [21], [23], [24].

*Angiostrongylus cantonensis* was first documented in Guangzhou (Canton), China in 1935 [25]. During the past 50 years, the parasite has spread from Southeast Asia to over 30 countries worldwide [26], [27]. There have been more than 2,800 cases of *A. cantonensis* infection in humans worldwide with 116 cases involving U.S. citizens [27], as well as numerous infections in other animals. Given the rapid dispersal of the parasite and the health implications for humans and wildlife, there is a need to determine the potential distribution of *A. cantonensis*.

To our knowledge, no global model for the current and potential distribution of *A. cantonensis* has been published. Although Lv et al. [22] published a comprehensive distribution of *A. cantonensis* within China, their model did not examine the potential distribution worldwide. The aim of this study was to use Maxent modeling to determine the maximum range distribution for the parasite globally and predict the potential future distribution of *A. cantonensis* under Intergovernmental Panel on Climate Change (IPCC) climate change scenarios.

## Methods

**Figure 1:**
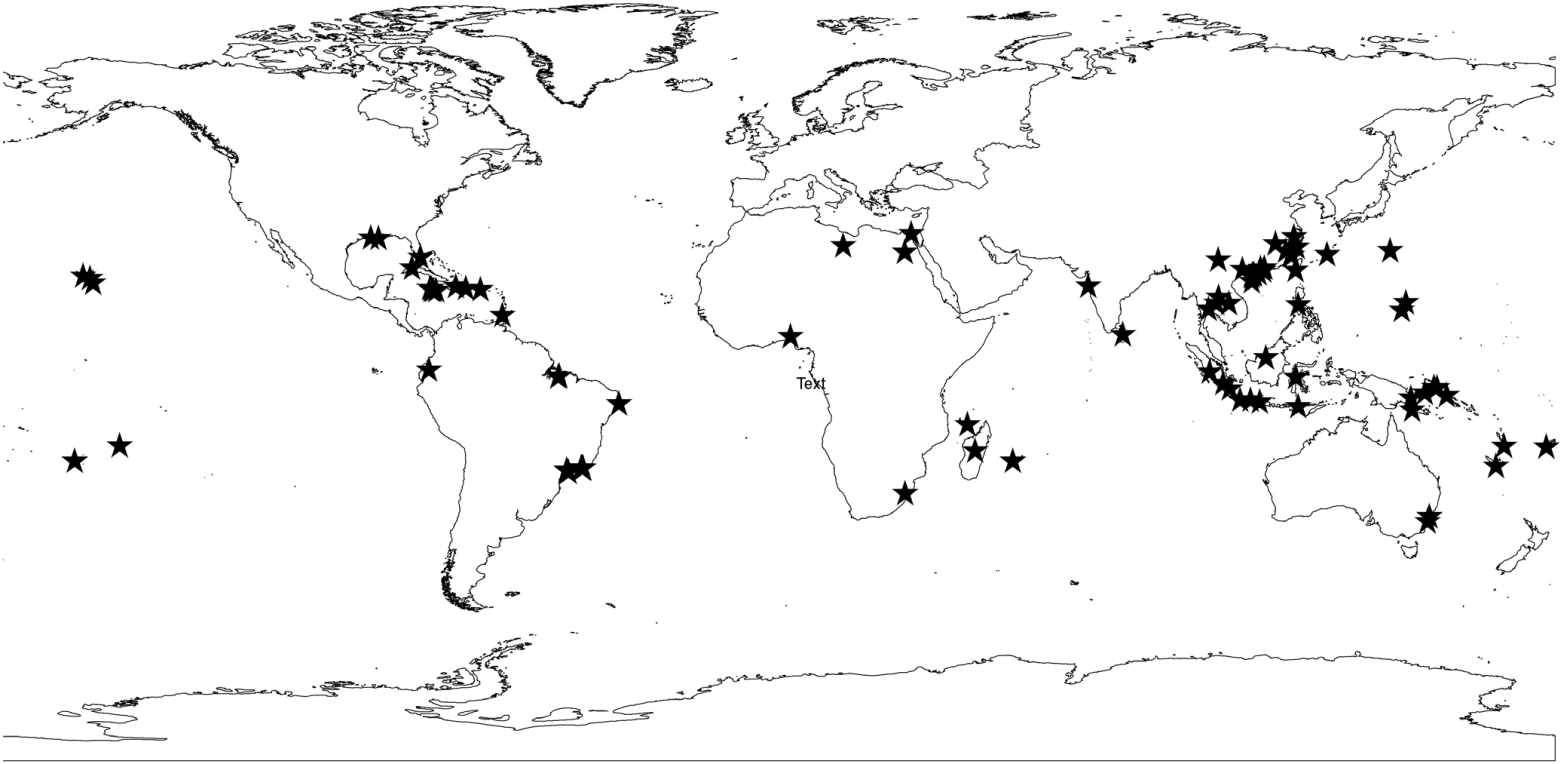
Current distribution of *A. cantonensis* based on locality data (n = 86) obtained from the literature.

Maxent was used to model the current and projected distribution of *A. cantonensis*. Documented occurrences of *A. cantonensis* were collected from published records. Records that met one or more of the following criteria were incorporated into our models: 1) documentation of the parasite in accepted endemic areas; 2) multiple cases of human infection (3 or more) within an area; 3) reports of the parasite found in intermediate or definitive hosts (with ≥ 3% prevalence) (Table S1). A total of 86 locations were included (Figure 1). Elevation and 19 climate variables were downloaded from WorldClim [28] with a resolution of 5 arc-minutes (100 km^2^; Table S2). All variables were included in the model initially. However, only the variables with the highest gain independent of others (Fig. S1) were retained, as these variables accounted for the greatest amount of the observed variation. In addition, the environmental variables that lowered the training gain the greatest when omitted were retained (Fig. S2), as these variables contained the most unique information. These variables were then retained for high multicollinearity (|r|>0.8) [29]. Additionally, Akaike’s information criterion for small sample correction (AICc) was used to evaluate the regularization of the models and to avoid overfitting [30]. All possible combinations of the variables that did not exhibit high multicollinearity were examined. Ten-fold cross-validation was used and receiver operating characteristic (ROC) curves were created by plotting sensitivity vs. 1–specificity to evaluate the accuracy of the resulting model. The area under the curve (AUC) was used to evaluate models. Models with an AUC score of 0.5 indicated a model preforming no better than random, while models with AUC score of 1 indicated a perfect model [11], [31]. However, AUC scores are not without limitations [32], [33] and should be used in conjunction with other model evaluation methods [34]. Consequently, we used AICc scores and model weights along with AUC scores to determine the model that best describes the current distribution of *A. cantonensis*.

IPCC 5 data for future climate conditions for the 2050s and 2070s were obtained from the International Centre for Tropical Agriculture (CIAT) [35] to project the potential future distribution of *A. cantonensis* at 5 arc-minutes (100 km^2^). Eleven IPCC models under four Representative Concentration Pathways (RCPs) (2.6, 4.5, 6.0, and 8.5) for the 2050s and 2070s were evaluated: BCC-CSM1-1, CCSM4, GISS-E2-R, HadGEM2-AO, HadGEM2-ES, IPSL-CM5A-LR, MIROC-ESM-CHEM, MIROC-ESM, MIROC5, MRI-CGCM3, and NorESM1-M [35]. The eleven models for each RCP scenario were then averaged to produce a total of 8 future models (four for the 2050s and four for the 2070s). We present our results as mean ± standard deviation. The change in area for each RCP during the 2050s and 2070s was calculated for highly suitable habitat (>50%) and total suitable habitat (>11.8%). We determined total suitable habitat using a 5% omission rate.

## Results

**Figure 2:**
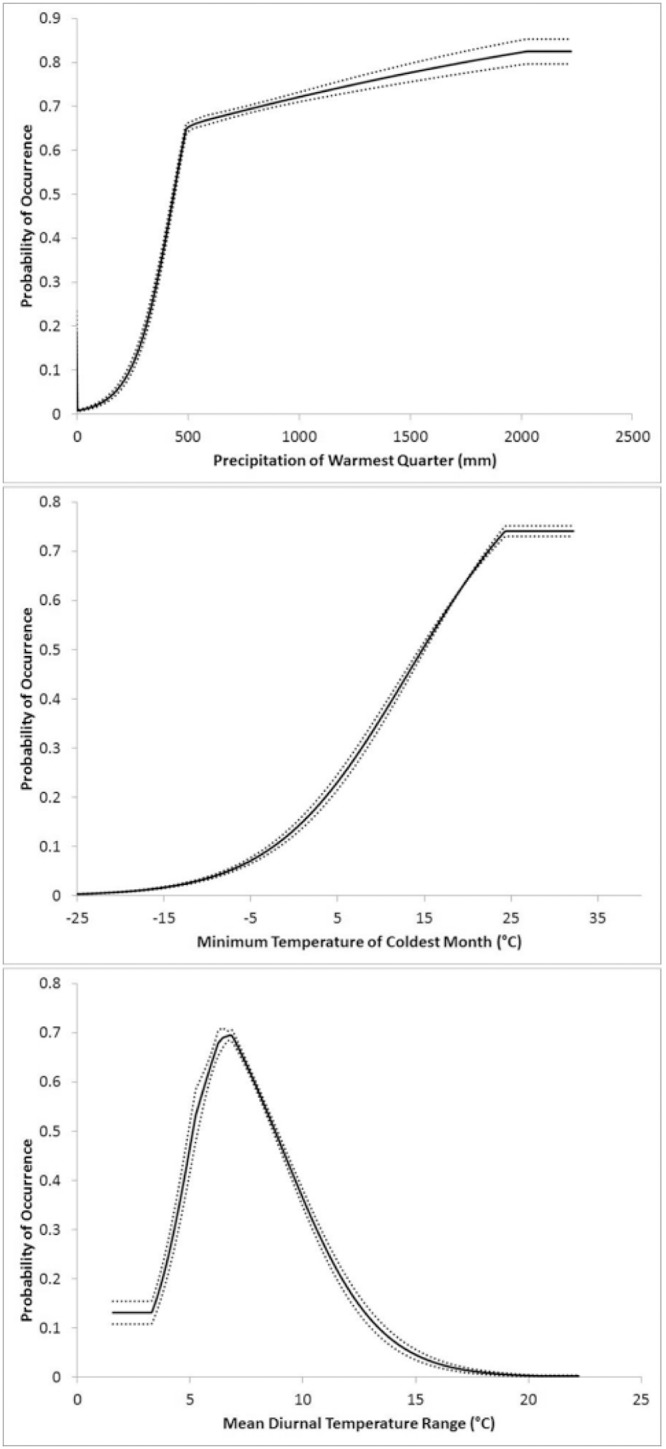
Probability of *A. cantonensis* presence in response to ecogeographical variables in the best-fit model.

**Figure 3:**
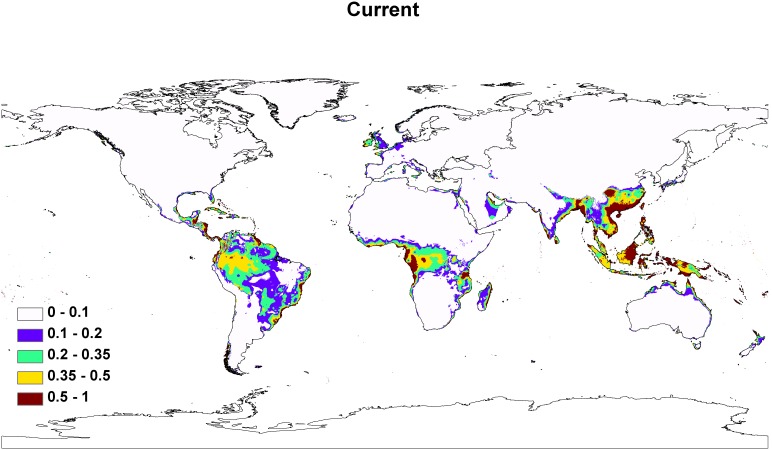
The Maxent model of the projected current distribution for *A. cantonensis.*

The best model (i.e., the model with the lowest small sample corrected variant of the AICc score) included three environmental variables: mean diurnal temperature range (BIO 2), minimum temperature of coldest month (BIO 6), and precipitation of warmest quarter (BIO 18; Table 1). The AUC was 0.945±0.029 for this model. Figure 2 displays suitability in response to the three variables. Areas that were predicted to have suitability >50% had a mean diurnal temperature range of 5.13–8.76°C, a minimum temperature of the coldest month of 14.79–32.17°C, and precipitation of the warmest quarter of 438.37–2,224.20 mm (Fig. 2). Areas with >50% suitability were found primarily in tropical areas (Fig. 3), including the reported native range in southeast Asia. When examining total suitable habitat of >11.8%, we found an increase in area futher north and south of the equator, extending into Europe and to New Zealand (Fig. 3).

**Table 1:** Comparison of the top three possible models.

Variables in Model	Log Likelihood*	AICc scores	ΔAICc	wAICc	Mean AUC
BIO 2, BIO 6, BIO 18	−1054.82	2152.646731	0	0.9987	0.945
BIO 2, BIO 6, BIO 15, BIO 18	−1045.22	2166.226719	13.579988	0.00112	0.946
BIO 2, BIO 6, BIO 12, BIO 18	−1047.25	2170.297934	17.6512034	0.00015	0.946

*Log-likelihood is the natural log of the probability of the data given in the model. AICc is a corrected AIC score, used for a small sample size by increasing the cost for each parameter. Delta AICc is the difference between the model with the lowest score (the “best” model) and the AICc score for each model. The model weight (wAICc) is the relative likelihood for each model, divided by the total relative likelihood for all models that were considered. AUC (area under the curve) is a measure of the accuracy of the model.

**Figure 4:**
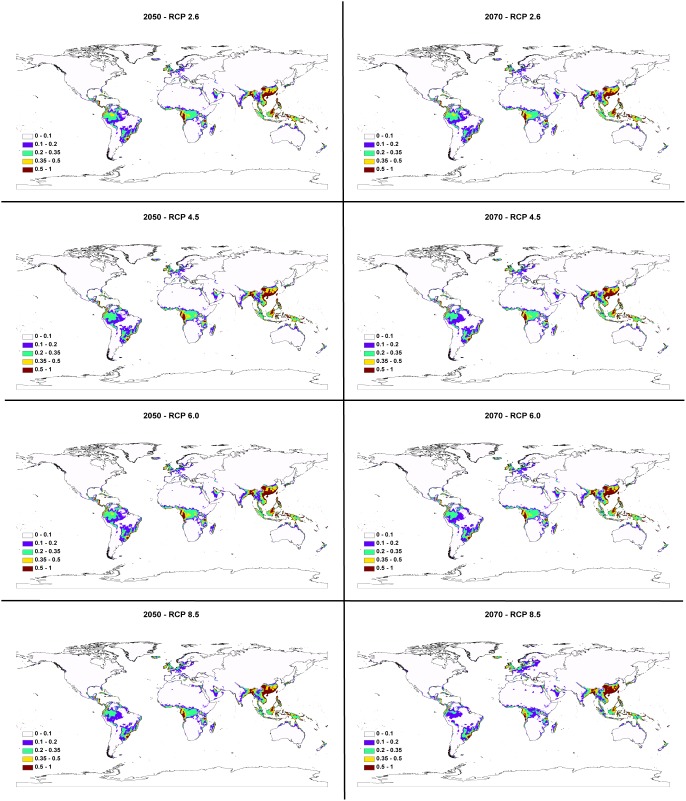
Comparison of model runs for *A. cantonensis*.

**Figure 5:**
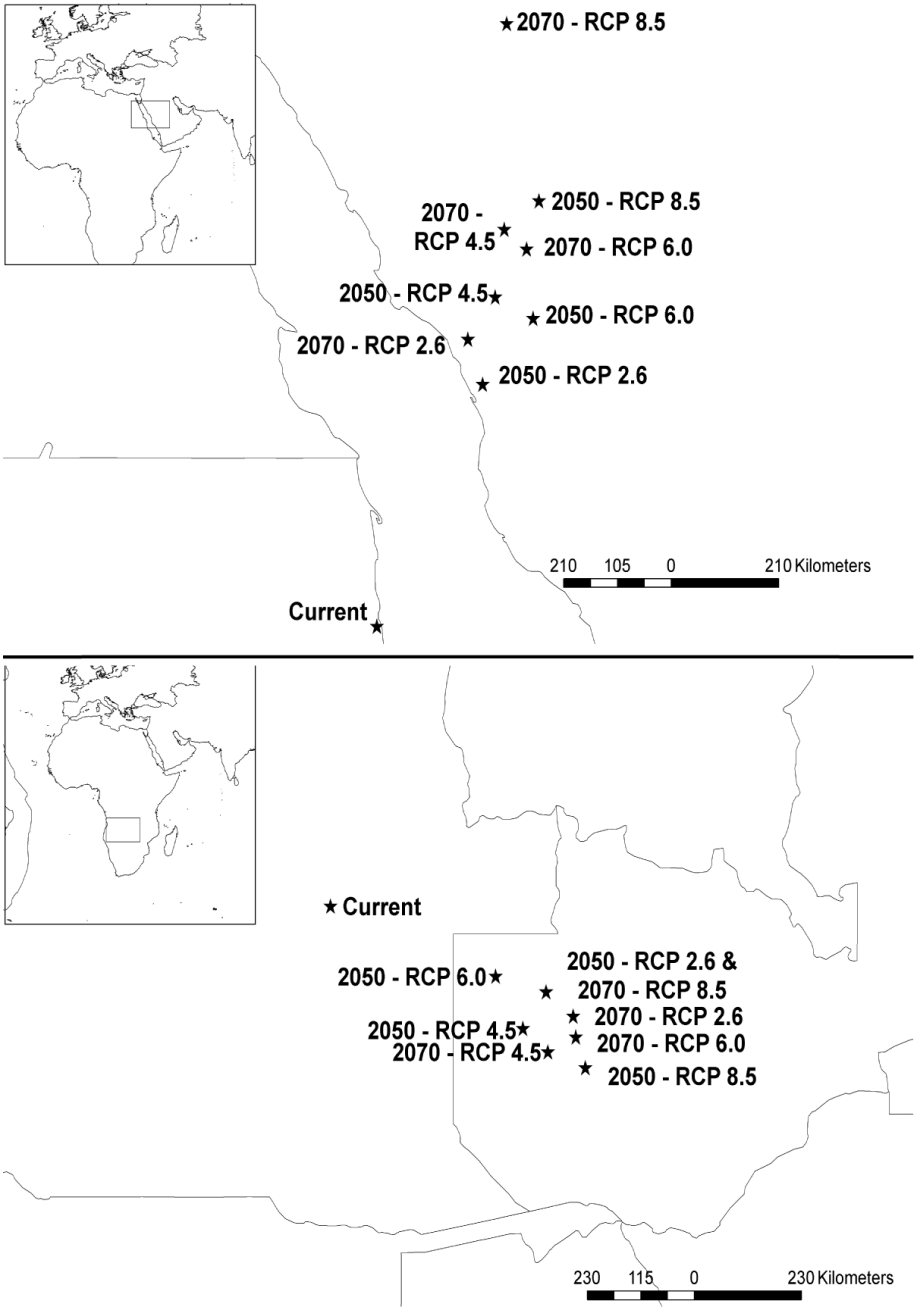
Northern and Southern Hemisphere centroids (indicated by stars) for all scenarios.

Areas with suitable climatic conditions for *A. cantonensis* are predicted to decline by the 2050s and the 2070s under all four RCP scenarios (Fig. 4). Currently, 6,160,942.17 km^2^ are highly suitable (i.e. >50% chance of suitability) and 33,432,536.52 km^2^ may be considered suitable (i.e. >11.8% chance of suitability). However, by the 2050s the amount of highly suitable habitat is expected to decrease to 5,196,405.69–5,504,137.11 km^2^, with 84.34–89.34% of the area in common with the current model (Table 2). The total habitat suitability also indicates an overal decrease to 32,577,236.91–33,265,264.86 km^2^ with 89.60–99.50% of the area in common with the current model (Table 3). By the 2070s, the area of highly suitable habitat will further decline in all four RCP scenarios, ranging from 5,168,469.42–5,353,298.55 km^2^, (Table 2). The four models based on RCP scenarios had 83.89–86.89% of the area shared with the current model (Table 2). When examining total suitable habitat, the future models had 97.00–98.45% of the area in common with the current model (Table 3). The centroid (geometric center of the species range) for the northern hemisphere is predicted to shift northeast 440.27–762.00 km by the 2050s, a rate of 88.05–152.40 km per decade (Table 4, Fig. 5). By the 2070s, the centroid is expected to continue to shift to the north or northeast by 499.82–1,027.87 km, a rate of 71.40–146.84 km per decade. In the southern hemisphere, the centroid is expected to shift southeast and east-southeast 369.34–610.53 km by the 2050s for all scenarios at a rate of 73.87–122.11 km per decade (Table 4, Fig. 5). By the 2070s, the centroid is predicted to shift east-southeast 479.80–566.90 km, a rate of 68.54–80.99 km per decade (Table 4). The only exception is that the centroid for the RCP 2.6 scenario is expected to shift northward by the 2070s from the 2050s centroid but remain east-southeast of the current centroid (Fig. 5).

**Table 2:** Total area predicted to have >50% probability of suitable habitat conditions for *A. cantonensis* under each climate change scenario.

Scenario	Area (km^2^)	% change in area	Area common to current (km^2^)	% of current distribution retained
Current	6160942.17			
2050s–26	5357536.56	−13.04%	4665876.03	86.96%
2050s–45	5387894.55	−12.55%	4432958.46	87.45%
2050s–60	5504137.11	−10.66%	4517113.23	89.34%
2050s–85	5196405.69	−15.66%	4023687.78	84.34%
2070s–26	5353298.55	−13.11%	4639496.58	86.89%
2070s–45	5235326.19	−15.02%	4176861.57	84.98%
2070s–60	5339287.17	−13.34%	4108620.96	86.63%
2070s–85	5168469.42	−16.11%	3387207.87	83.89%

**Table 3:** Total area predicted to have >11.8% probability of suitable habitat conditions for *A. cantonensis* under each climate change scenario.

**Scenario**	**Area (km^2^)**	**% change in area**	**Area common to current (km^2^)**	**% of current distribution retained**
Current	33432536.52			
2050s–26	32577236.91	−2.56%	29954427.66	89.60%
2050s–45	33078100.5	−1.06%	30045069.18	99.50%
2050s–60	33265264.86	−0.50%	29835763.38	97.63%
2050s–85	32658191.55	−2.32	28675327.05	97.85%
2070s–26	32713026.21	−2.15%	29997067.23	98.25%
2070s–45	32847518.16	−1.75%	29113052.94	98.45%
2070s–60	32914374.93	−1.55	28863269.82	98.45%
2070s–85	32428906.56	−3.00%	26600172.48	97.00%

**Table 4:** Summary of the distance from each projected centroid for each RCP scenario (2.6, 4.5, 6.0, and 8.5) to the current centroid as well as the rate per decade.

Scenario	Distance (km) to current centroid	Rate of km per decade
Current North		
2050s–2.6	440.27 (NE)	88.05 km/decade
2050s–4.5	583.41 (NE)	116.68 km/decade
2050s–6.0	580.02 (NE)	116.00 km/decade
2050s–8.5	762.00 (NE)	152.40 km/decade
2070s–2.6	499.82 (NNE)	71.40 km/decade
2070s–4.5	695.15 (NE)	99.31 km/decade
2070s–6.0	679.03 (NNE)	97.00 km/decade
2070s–8.5	1027.87 (NNE)	146.84 km/decade
Current South		
2050s–2.6	479.80 (ESE)	95.96 km/decade
2050s–4.5	462.34 (SE)	92.47 km/decade
2050s–6.0	369.34 (SE)	73.87 km/decade
2050s–8.5	610.53 (ESE)	122.11 km/decade
2070s–2.6	547.55 (ESE)	78.22 km/decade
2070s–4.5	529.11 (ESE)	75.59 km/decade
2070s–6.0	566.90 (ESE)	80.99 km/decade
2070s–8.5	479.80 (ESE)	68.54 km/decade

## Discussion

The effects of global climate change are hypothesized to influence disease range expansions (via pathogen spread) and indirect expansions (via reservoirs, hosts, or vector range expansions). This will increase the frequency of disease outbreaks and expand the pool of at-risk populations [16], [36], [37]. Hales et al. [38], predict an increase in land area compatible for Dengue fever transmission by 2085, with 50–60% of the world’s population at risk. Within North America, leishmaniasis reservoirs and vectors are predicted to undergo a range expansion northward, leading to greater human exposure [18]. However, an emerging picture of the effects of global climate change on disease is that an increase in habitat suitability in one area will be balanced by decreased suitability elsewhere, leading to a range shift or reduction [4], [39]. Although the proximate expansion of *A. cantonensis* into new suitable regions continues via introduction of definitive and intermediate hosts, our findings predict an ultimate decline of up to 16% in area of highly suitable bioclimatic habitat and up to a 3% decline in total suitable bioclimatic habitat by the 2070s.

The global model for the present distribution of *A. cantonensis* predicts that the most suitable habitat is located near the equator in tropical to subtropical regions. Three bioclimatic variables were found to contribute the most to predicting the potential distribution of the parasite: minimum temperature of the coldest month, minimum diurnal temperature range, and precipitation of the warmest quarter. Under all IPCC climatic scenarios, our models predict a shift in the distribution of suitable habitat for *A. cantonensis* in the Northern and Southern hemispheres. Under all RCP climatic scenarios, a shift in the distribution of the parasite is expected to occur north and east in the Northern hemisphere by the 2050s (range = 88.05–152.40 km per decade), continuing through the 2070s (range = 71.40–146.84 km per decade). The shift in the distribution of the parasite in the Southern hemisphere under all climatic scenarios is predicted to occur southeastward by the 2050s (range = 73.87–122.11 km per decade) and through the 2070s (range = 68.54–80.99 km per decade). Although there have been no endemic reports of *A. cantonensis* within Europe, all four models suggest an increase in suitable habitat for *A. cantonensis* within Europe while simultaneously showing an overall decline in global suitability. This potential range shift into Europe is most likely due to a predicted increase in the minimum temperature of the coldest month, which demonstrates the need for additional monitoring programs within Europe. These programs should include long-term surveying or screening for the parasite within definitive, paratenic, and intermediate hosts. Furthermore, an increase in public health programs targeted at awareness of the parasite and its transmission will be essential in deterring an increase in human infection.

Temperature and precipitation are environmental variables that significantly influence the distribution of *A. cantonensis*. Because temperature plays a critical role in influencing biological processes [4] it will likely have a significant impact on pathogens, infectious disease hosts, vectors and reservoirs. As global temperatures rise (IPCC), there is increased potential for vector-borne diseases and pathogens to spread and/or increase in severity [40]–[43]. Increases in temperature can speed the rate of development for some malarial protozoa, increasing the risk of transmission from mosquito to host [44]. However, the positive association between temperature and pathogen transmission might be offset by a pathogen’s total bioclimatic requirements for survival. If such requirements are not met, host, vector, and/or pathogen mortality might increase. Similarly, increased temperatures might initially promote the spread and occurrence of *A. cantonensis*. However, with an expected temperature increase of 1.4°C–5.8°C from 1990 to 2100 [43] in areas where the bio-climatic norm exceeds an ecologically critical threshold temperature, resources needed to support parasitic growth and reproduction may become increasingly limited [4], [45]. Such demands could restrict the distribution of the parasite to areas with sufficient resources, potentially limiting disease incidence.

Global climate change is expected to increase the risk of intense precipitation and increased humidity in some regions, whereas other regions will experience extreme drought [35]. The effects of climatic variability in precipitation might induce the emergence of diseases in new areas or intensify infection rates of endemic pathogens. In several cases, disease occurrence has been demonstrated to be positively [41], [46]–[49] associated with rainfall. Alternatively, regions experiencing drought might negatively impact pathogen viability. Many parasites having intermediate hosts, such as *A. cantonensis,* require moist or wet environments for development and survival. Without sufficient precipitation, the distribution of the parasite might become more restricted, thereby decreasing the risk of transmission.

As *A. cantonensis* continues to spread, health complications in both humans and wildlife are expected to increase. Following introduction into a new area, *A. cantonensis* quickly infects and causes illness in humans, domestic animals and wildlife [19], [50]–[52]. Infected humans are often hospitalized with eosinophilic meningitis, and might also experience extraocular muscular paralysis [53]. In wildlife, *A. cantonensis* can cause a variety of symptoms (e.g. lethargy, limb paralysis) due to neurological invasion and might result in death [19], [50], [54]–[58]. To deter future outbreaks, *A. cantonensis* monitoring programs should be established worldwide, evaluating known definitive, intermediate and paratenic hosts and other wildlife. In addition, increased public awareness of the parasite, and the means by which it is transmitted, may lead to a lower incidence of infection.

These results provide the first global perspective of higher-risk areas for *A. cantonensis* colonization and overall changes in habitat suitability. The methodology employed here has been applied broadly to other studies on global climate change. More recently the application of ENM in evaluating disease distribution, risks and spread [12], [13], [17], [18], [59], [60] has proven useful. By identifying and documenting the distributions and ontogenetic requirements of known hosts (e.g., *Rattus* sp., molluscs) and conducting field surveys for the parasite, future studies might provide a much improved and conservative representation of the current and future range of *A. cantonensis*.

## Supporting Information

Figure S1 **Jackknife of regularized training gain for individual bioclimatic variables.**
(TIF)

Figure S2 **Jackknife of regularized training gain omitting each bioclimatic variable is shown.**
(TIF)

Table S1 **Locality Data.**
(DOCX)

Table S2 **Bioclimatic variables used in the construction of the niche models.**
(DOCX)
