# Supplementary figures and images for: Global Decline in Suitable Habitat for Angiostrongylus ( = Parastrongylus) cantonensis: The Role of Climate Change
Source: PLoS One. 2014 Aug 14;9(8):e103831. doi: 10.1371/journal.pone.0103831 (PMC4133392; doi:10.1371/journal.pone.0103831)

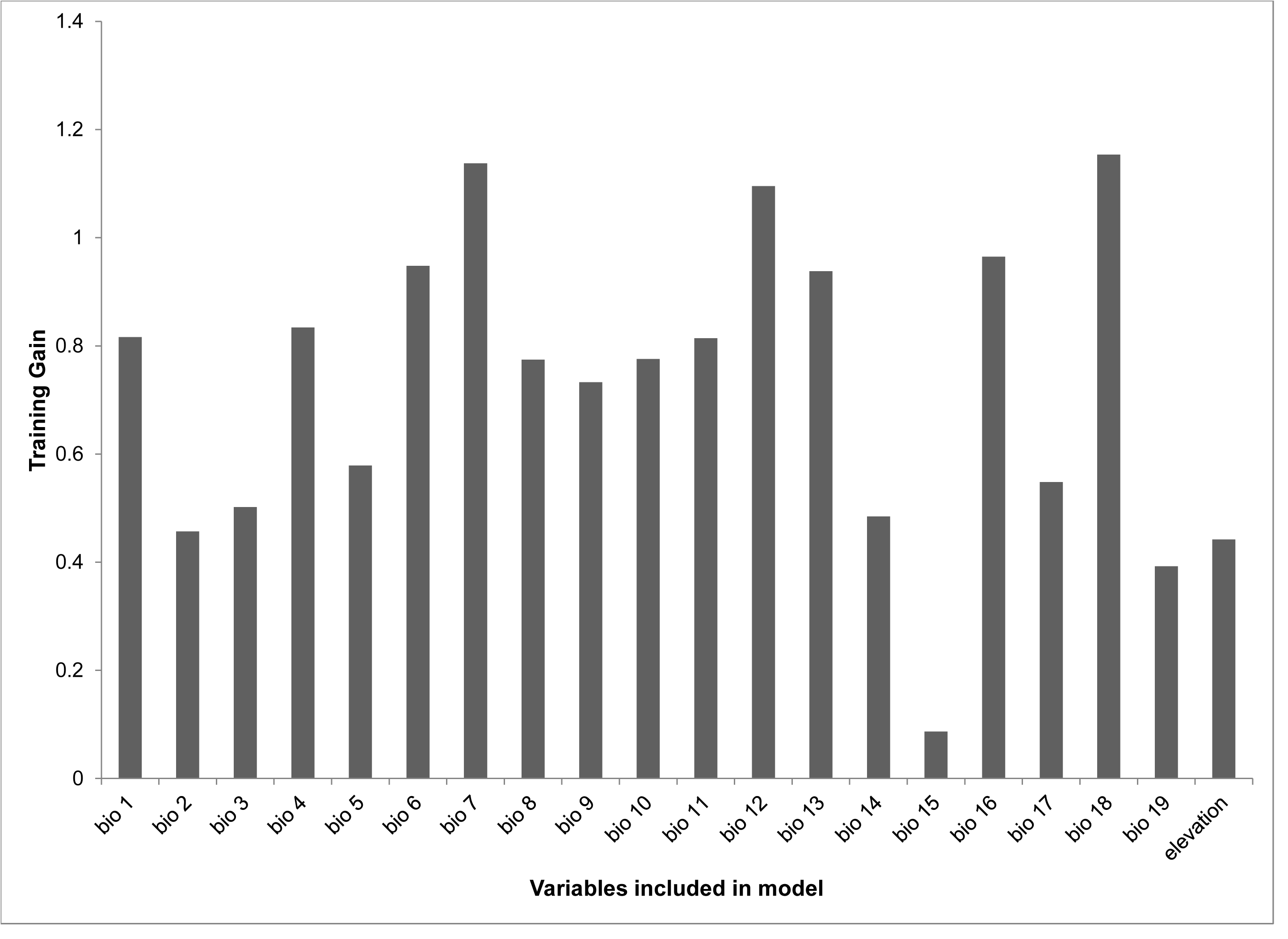

Supplement: Figure S1 — Jackknife of regularized training gain for individual bioclimatic variables. (TIF) [file pone.0103831.s001.tif]

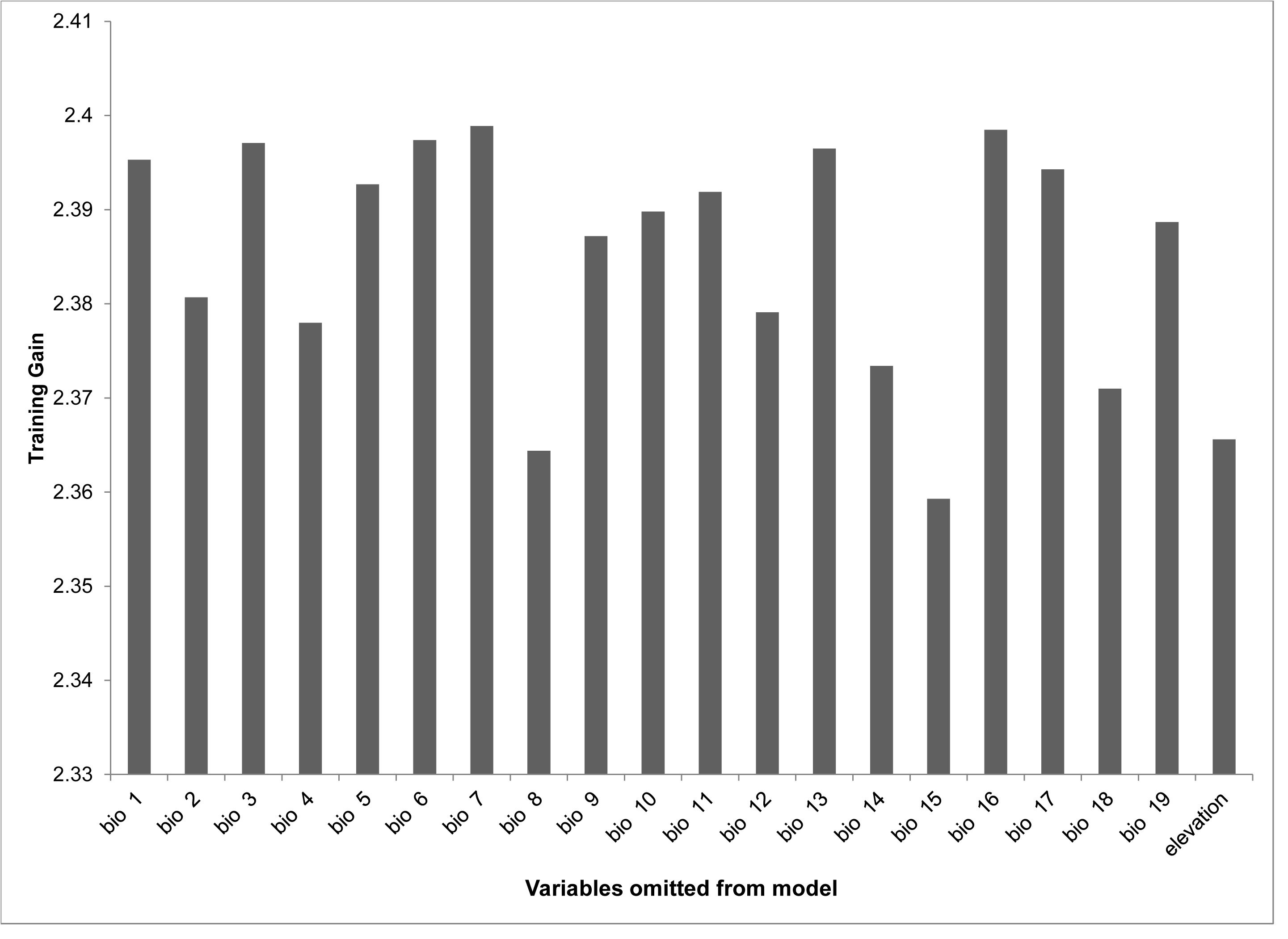

Supplement: Figure S2 — Jackknife of regularized training gain omitting each bioclimatic variable is shown. (TIF) [file pone.0103831.s002.tif]
