# Supplementary material for: Global Decline in Suitable Habitat for Angiostrongylus ( = Parastrongylus) cantonensis: The Role of Climate Change
Source: PLoS One. 2014 Aug 14;9(8):e103831. doi: 10.1371/journal.pone.0103831 (PMC4133392; doi:10.1371/journal.pone.0103831)
Supplement: Table S1 — Locality Data. (DOCX) [file pone.0103831.s003.docx]

**Table S1: Locality Data**

| **Geographic Region:** | **Location:** | **Latitude:** | **Longitude:** | **Literature Source:** |
| --- | --- | --- | --- | --- |
| Africa – Eastern | Madagascar | -18.766947 | 46.869108 | 1 |
| Africa – Eastern | Mayotte | -12.827436 | 45.166281 | 2 |
| Africa – Eastern | Réunion Island | -21.115142 | 55.536383 | 3 |
| Africa – Northern | North Siani, Egypt | 31.024842 | 32.287325 | 4 |
| Africa – Northern | Egypt (Cairo) | 26.820553 | 30.802497 | 5 |
| Africa – Western | Ile-Ife, Nigeria | 7.458336852 | 4.541676291 | 6 |
| Africa - South | KwaZulu-Natal | -28.54166503 | 30.875011 | 7 |
| Asia – Eastern | Changle, Fujian, China | 25.963119 | 119.523383 | 8 |
| Asia – Eastern | Fuzhou, Fujian, China | 26.074508 | 119.296494 | 9 |
| Asia – Eastern | Jianou, Fujian, China | 27.022728 | 118.304989 | 10 |
| Asia – Eastern | Zhanjiang, Guangdong, China | 21.267631 | 110.3609 | 11 |
| Asia – Eastern | Maoming, Guangdong, China | 21.663 | 110.925456 | 11 |
| Asia – Eastern | Jiangmen, Guangdong, China | 22.578739 | 113.0819 | 11 |
| Asia – Eastern | Yangjiang, Guangdong, China | 21.857958 | 111.982233 | 11 |
| Asia – Eastern | Zhaoqing, Guangdong | 23.047192 | 112.465092 | 11 |
| Asia – Eastern | Huazhou, Guangdong | 21.664936 | 110.640369 | 12 |
| Asia – Eastern | Guangxi, China | 22.815478 | 108.327544 | 13,14 |
| Asia – Eastern | Canton/Guangzhou, China | 23.129164 | 113.264436 | 15 |
| Asia – Eastern | Hainan, China | 20.017378 | 110.349228 | 16 |
| Asia – Eastern | Jiangxi, China | 28.674425 | 115.909175 | 10 |
| Asia – Eastern | Kunming, Yunnan, China | 25.037722 | 102.722203 | 17 |
| Asia – Eastern | Zhejiang, China | 27.994267 | 120.699367 | 18 |
| Asia – Eastern | Zhejiang, China | 30.266292 | 120.153822 | 19 |
| Asia – Eastern | Bonin (Ogasawara) Islands | 27.094294 | 142.192036 | 20 |
| Asia – Eastern | Okinawa, Japan | 26.2124 | 127.680933 | 21 |
| Asia – Eastern | Taiwan | 22.655789 | 120.470289 | 22 |
| Asia – Eastern | Quezon City, Phillipines | 14.676042 | 121.0437 | 23 |
| Asia – Southern | India | 19.017614 | 72.856164 | 24, 25 |
| Asia – Southern | Sri Lanka | 7.873053 | 80.771797 | 24 |
| Asia – South-Eastern | Sarawak, Borneo | 2.607342 | 113.648944 | 26 |
| Asia – South-Eastern | Central Java | -7.150975 | 110.140258 | 27 |
| Asia – South-Eastern | West Java | -7.090883 | 107.668861 | 27 |
| Asia – South-Eastern | East Java | -7.536064 | 112.238403 | 27 |
| Asia – South-Eastern | Lampung | -4.558586 | 105.406808 | 27 |
| Asia – South-Eastern | East Nusa Tenggara | -8.657383 | 121.079369 | 27 |
| Asia – South-Eastern | South Sumatra | -3.319436 | 103.9144 | 27 |
| Asia – South-Eastern | West Sumatra | -0.739939 | 100.800006 | 27 |
| Asia – South-Eastern | Khon kaen, Thailand | 16.45833732 | 102.7916814 | 28 |
| Asia – South-Eastern | Bangkok, Thailand | 13.716731 | 100.54064 | 29, 30 |
| Asia – South-Eastern | Korat, Thailand | 15.017228 | 102.316944 | 29 |
| Asia – South-Eastern | Ubon, Thailand | 15.072708 | 105.219481 | 29 |
| Asia – South-Eastern | North Sulawesi | -1.847908 | 120.527911 | 27 |
| Australia | Beecroft Peninsula, Australia | -35.082594 | 150.813367 | 31 |
| Australia | Sydney, Australia | -33.89175 | 151.199522 | 32 |
| Caribbean | Santo Domingo, Dominican Republic | 18.499997 | -69.983331 | 33, 34 |
| Caribbean | Cuba | 23.115292 | -82.389031 | 35, 36, 37 |
| Caribbean | Haiti | 18.971186 | -72.285214 | 38 |
| Caribbean | Black River, Jamaica | 18.03085 | -77.852158 | 39 |
| Caribbean | Kingston, Jamaica | 17.992731 | -76.792008 | 39 |
| Caribbean | Lucea, Jamaica | 18.44275 | -78.178628 | 39 |
| Caribbean | Mandeville, Jamaica | 18.039661 | -77.513283 | 39 |
| Caribbean | Montego Bay, Jamaica | 18.466667 | -77.916667 | 39 |
| Caribbean | Puerto Rico | 18.220833 | -66.59015 | 40 |
| Caribbean | Grenada, West Indies | 12.262775 | -61.604172 | 41 |
| Europe – Southern | Tenerife, Canary Islands | 28.291564 | 16.629131 | 42, 43 |
| Melanesia | Viti Levu, Fiji | -17.848319 | 178.011847 | 44, 45 |
| Melanesia | Noumea, New Caledonia | -22.2758 | 166.458 | 46 |
| Melanesia | Efate, New Hebride | -17.735261 | 168.321731 | 47 |
| Melanesia | Bougainville, Papua New Guinea | -6.0536 | 155.190681 | 48 |
| Melanesia | Kimbe, Papua New Guinea | -5.550433 | 150.142808 | 48 |
| Melanesia | Lae, Papua New Guinea | -6.723669 | 146.990906 | 48 |
| Melanesia | New Ireland, Papua New Guinea | -4.285325 | 152.920592 | 48 |
| Melanesia | Port Moresby, Papua New Guinea | -9.481553 | 147.190242 | 48 |
| Melanesia | Rabaul, Papua New Guinea | -4.196161 | 152.172961 | 48 |
| Micronesia | Guam | 13.444275 | 144.793731 | 49 |
| Micronesia | Saipan | 15.1778 | 145.750967 | 49 |
| Northern America | Miami, Florida | 25.611517 | -80.397781 | 50, 51 |
| Northern America | Big Island, Hawaii | 19.693236 | -155.537814 | 52 |
| Northern America | Honolulu, Hawaii | 21.301281 | -157.860656 | 52 |
| Northern America | Maui, Hawaii | 20.808581 | -156.319975 | 52 |
| Northern America | New Orleans, Louisiana | 29.952858 | -90.071242 | 53, 54 |
| Northern America | New Iberia, Louisiana | 30.003536 | -91.815367 | 53 |
| Polynesia | Rarotonga, Cook Islands | -21.229236 | -159.77635 | 55 |
| Polynesia | French Polynesia (Tahiti) | -17.650919 | -149.426042 | 56 |
| South America | São Mateus, SP, Brazil | -23.594494 | -46.474553 | 57 |
| South America | Jundiaí, SP, Brazil | -23.215122 | -46.880469 | 57 |
| South America | Pirituba, SP, Brazil | -23.479 | -46.723122 | 57 |
| South America | São Gonçalo, RJ, Brazil | -22.825 | -43.034167 | 57 |
| South America | Encantado, RJ, Brazil | -22.895833 | -43.302222 | 57 |
| South America | Caju, RJ, Brazil | -22.882814 | -43.218708 | 57 |
| South America | Niterói, RJ, Brazil | -22.898642 | -43.131658 | 57 |
| South America | Queimados, RJ, Brazil | -22.715333 | -43.568333 | 57 |
| South America | Marituba, PA, Brazil | -1.616389 | -48.580389 | 57 |
| South America | Jurunas, PA, Brazil | -1.792861 | -48.823556 | 57 |
| South America | Olinda, PE, Brazil | -8.008958 | -34.852208 | 57 |
| South America | Quito, Ecuador | -0.180653 | -78.467839 | 58 |

TABLE REFERECES

1. Breuil J, Coulanges P (1982) Note sur *Angiostrongylus cantonensis* à Madagascar. Arch Inst Pasteur Madagascar 50: 35-38.

2. Graber D, Hebert JC, Jaffar-Bandjee MC, Alessandri JL, Combes JC (1999) Severe forms of eosinophilic meningitis in infants of Mayotte. Apropos of 3 cases. Bull Soc Pathol Exot 92: 164-166.

3. Graber D, Jaffar MC, Attali T, Poisson J, Renouil M, et al. (1996). *Angiostrongylus* in the infant at Reunion and Mayotte. Apropos of 3 cases of eosinophilic meningitis including 1 fatal radiculomyeloencephalitis with hydrocephalus. Bull Soc Pathol Exot 90: 331-332.

4. Ibrahim, MM (2007) Prevalence and intensity of *Angiostrongylus cantonensis* in freshwater snails in relation to some ecological and biological factors. Parasite 14: 61-70.

5. Yousif F, Ibrahim A. (1978) The first record *ofAngiostrongylus cantonensis* from Egypt. Zeitschrift für Parasitenkunde 56: 73-80.

6. Sowemimo OW, Asaolu SP (2004) The prevalence of *Angiostrongylus cantonensis* (Chen) in *Archachatina marginata* (Swainson) in Ile-Ife, Nigeria. Ife Journal of Science 6: 105-107.

7. Archer CE, Appleton CC, Mukaratirwa S, Hope KJ (2011) The rat lung-worm *Angiostrongylus cantonensis:* A first report in South Africa. S Afr Med J 101: 174-175.

8. Lin JX, Li YS, Zhu K, Chen BJ, Cheng YZ, et al. (2003) Epidemiological study on group infection of *Angiostrongylus cantonensis* in Changle City. Zhongguo Ji Sheng Chong Xue Yu Ji Sheng Chong Bing Za Zhi 21: 110-112.

9. Luo B, Chen JY, Ye DG, Liu BR, Zheng P (2005). A survey on epidemic focus of *Angiostrongylus cantonensis* in Fuzhou. Zhongguo Ren Shou Gong Huan Bing Za Zhi 21: 829-830.

10. Zhang Y, Lv S, Yang K, Liu HX, Hu L, et al. (2009) The first national survey on natural nidi of *Angiostrongylus cantonensis* in China. Zhongguo Ji Sheng Chong Xue Yu Ji Sheng Chong Bing Za Zhi 27: 508-512.

11. Chen D, Zhang Y, Shen H, Wei Y, Huang D, et al. (2011) Epidemiological survey of *Angiostrongylus cantonensis* in the west-central regions of Guangdong province, China. Parasitol Res 109: 305-314.

12. Deng ZH, Zhang QM, Huang SY, Jones JL (2012) First provincial survey of *Angiostrongylus cantonensis* in Guangdong Province, China. Trop Med Int Health 17: 119-122.

13. Lv S, Zhang Y, Steinmann P, Zhou X-N (2008) Emerging angiostrongyliasis in mainland China. Emerg Infect Dis 14: 161-164.

14. Zhang HM, Tan YG, Li XM, Ruan TQ, Zhou XN, et al. (2007) Survey on the infectious focus of *Angiostrongylus cantonensis* in Guangxi. J Trop Dis Parasitol *5*: 79- 84.

15. Chen CX, He HF, Yin Z, Zhou JH, Li SQ, et al. (2012) Investigation on snails *Achatina fulica* and *Pomacea canaliculata* infected with *Angiostrongylus cantonensis* in Panyu region of Guangzhou City. Zhongguo Xue Xi Chong Bing Fang Zhi Za Zhi 24: 336-338.

16. Hu X, Du J, Tong C, Wang S, Liu J, et al. (2011) Epidemic status of *Angiostrongylus cantonensis* in Hainan island, China. Asian Pac J Trop Med 4: 275-277.

17. Wei LP, Zheng KW, Wei Y (2005) A report on clinically nursing 9 angiostrongyliasis patients. J Clin Nurs 4: 21–22.

18. Xue DY, Ruan YZ, Lin BC, Zheng RY, Fang JQ, et al. (2000). Epidemiological investigation on an outbreak of angiostrongyliasis cantonensis in Wenzhou. Zhongguo ji sheng chong xue yu ji sheng chong bing za zhi 18: 176-178.

19. Lin W, Wang XT (2004) Epidemiology of *Angiostrongylus cantonensis* in mainland. Zhongguo Ren Shou Gong Huan Bing Za Zhi 20: 1004-1007.

20. Tokiwa T, Hashimoto T, Yabe T, Komatsu N, Akao N, et al. (2013) First Report of *Angiostrongylus cantonensis* (Nematoda: Angiostrongylidae) Infections in Invasive Rodents from Five Islands of the Ogasawara Archipelago, Japan. PLoS One 8:e70729.

21. Tokiwa T, Harunari T, Tanikawa T, Komatsu N, Koizumi N, et al. (2012) Phylogenetic relationships of rat lungworm, *Angiostrongylus cantonensis*, isolated from different geographical regions revealed widespread multiple lineages. Parasitol Int 61: 431-436.

22. Tseng YT, Tsai HC, Sy CL, Lee SSJ, Wann SR, et al. (2011) Clinical manifestations of eosinophilic meningitis caused by *Angiostrongylus cantonensis*: 18 years’ experience in a medical center in southern Taiwan. J Microbiol Immunol Infect 44: 382-389.

23. Fontanilla IKC, Wade CM (2008) The small subunit (SSU) ribosomal (r) RNA gene as a genetic marker for identifying infective 3rd juvenile stage *Angiostrongylus cantonensis.* Acta Tropica 105: 181-186.

24. Panackel C, Cherian G, Vijayakumar K, Sharma RN (2006) Eosinophilic meningitis due to *Angiostrongylus cantonensis*. Indian J Med Microbiol 24: 220-221.

25. Renapurkar DM, Bhopale MK, Limaye LS, Sharma KD (1982) Prevalence of *Angiostrongylus cantonensis* infection in commensal rats in Bombay. J Helminthol 56: 345-349.

26. LiM BL (1970) Further studies of *Angiostrongylus cantonensis* in Kuching, Sarawak with special reference to the molluscan intermediate hosts. Southeast Asian J Trop Med Public Health 1: 112-116.

27. Carney WP, Stafford EE, Purnomo, Tanudjaja S (1978) Angiostrongyliasis in Indonesia: additional geographic and host occurrence records. Southeast Asian J Trop Med Public Health 9: 516-519.

28. Sawanyawisuth K, Takahashi K, Hoshuyama T, Sawanyawisuth K, Senthong V, et al. (2009) 698 Clinical Factors Predictive of Encephalitis Caused by *Angiostrongylus cantonensis*. Am J Trop Med Hyg 81: 698-701.

29. Punyagupta S, Bunnag T, Juttijudata P, Rosen L (1970) Eosinophilic meningitis in Thailand. Am J Trop Med Hyg 19: 950-958.

30. Witoonpanich R, Chuahirun S, Soranastaporn S, Rojanasunan P. (1991) Eosinophilic myelomeningoencephalitis caused by *Angiostrongylus cantonensis*: a report of three cases. Southeast Asian J Trop Med Public Health 22: 262-267.

31. Stokes VL, Spratt DM, Banks PB, Pech RP, Williams RL (2007) Occurrence of *Angiostrongylus species* (Nematoda) in populations of *Rattus rattus* and *Rattus fuscipes* in coastal forests of south-eastern Australia. Aust J Zool 55: 177-184.

32. Monks DJ, Carlisle MS, Carrigan M, Rose K, Spratt D, et al. (2005) *Angiostrongylus cantonensis* as a cause of cerebrospinal disease in a yellow-tailed black cockatoo (*Calyptorhynchus funereus*) and two tawny frogmouths (*Podargus strigoides*). J Avian Med Surg 19: 289-293.

33. Vargas M, Gomez Perez JD, Malek EA (1992) First record of *Angiostrongylus cantonensis* (Chen, 1935) (Nematoda: Metastrongylidae) in the Dominican Republic. Trop Med Parasitol 43: 253-255.

34. Rau C, Bialek R, Richter S, Lindner A (2006) Headache after a stay in the Dominican Republic. Dtsch Med Wochenschr 131: 1656-1659.

35. Aguiar PH, Morera P, Pascual J (1981) First record of *Angiostrongylus cantonensis* in Cuba. Am J Trop Med Hyg 30: 963-965.

36. Bärtschi E, Bordmann G, Blum J, Rothen M (2004). Eosinophilic meningitis due to *Angiostrongylus cantonensis* in Switzerland. Infection 32: 116-118.

37. Martinez-Delgado JF, Gonzalez-Cortinas M, Tapanes-Cruz TR, Ruiz-Mendez A (2000) Meningoencefalitis en Villa Clara (Cuba): estudio de 17 pacientes. Rev Neurol 31: 417-421.

38. Raccurt CP, Blaise J, Durette-Desset MC (2003) Presence of *Angiostrongylus cantonensis* in Haiti. Trop Med Int Health 8: 423-426.

39. Lindo JF, Waugh C, Hall J, Cunningham-Myrie C, Ashley D, et al. (2002) Ezoonotic *Angiostrongylus cantonensis* in rats and snails after an outbreak of human eosinophilic meningitis, Jamaica. Emerg Infect Dis 8: 324-326.

40. Andersen E, Gubler DJ, Sorensen K, Beddard J, Ash LR (1986) First report of *Angiostrongylus cantonensis* in Puerto Rico. Am J Trop Med Hyg 35: 319-322.

41. Chikweto A, Bhaiyai M, Maepherson CNL, DeAllie C, Pickney RD et al. (2009) Existence of *Angiostrongylus cantonensis* in rats (*Rattus norvegicus*) in Grenada, West

Indies. Vet Parasitol 162: 160-162.

42. Foronda P, Lopez-Gonzalez M, Miquel J, Torres J, Segovia M, et al. (2010) Finding of *Parastrongylus cantonensis* (Chen, 1935) in *Rattus rattus* in Tenerife, Canary Islands (Spain). Acta Trop 114: 123-127.

43. Martin-Alonso A, Foronda P, Quispe-Ricalde MA, Feliu C, Valladares B (2011) Seroprevalence of *Angiostrongylus cantonensis* in Wild Rodents from the Canary Islands. PLoS ONE 6: e27747.

44. Uchikawa R, Takagi M, Matayoshi S, Sato A (1984) The presence of *Angiostrongylus cantonensis* in Viti Levu, Fiji. J Helminthol 58: 231-234.

45. Wood G, Delamont S, Whitby M, Boyle R (1991) Spinal sensory radiculopathy due to *Angiostrongylus cantonensis* infection. Postgrad Med J 67: 70-72.

46. Ash LR (1976) Observations on the role of mollusks and planarians in the transmission of *Angiostrongylus cantonensis* infection to man in New Caledonia. Rev Biol Trop 24: 163-174.

47. Bowden DK (1981) Eosinophilic meningitis in the New Hebrides two outbreaks and two deaths. Am J Trop Med Hyg 30: 1141-1143.

48. Scrimgeour EM, Welch JS (1984) *Angiostrongylus cantonensis* in east New Britain, Papua New Guinea. Trans R Soc Trop Med Hyg 78: 774-775.

49. Alicata JE (1965) Biology and distribution of the rat lungworm, *Angiostrongylus cantonensis*, and its relationship to eosinophilic meningoencephalitis and other neurological disorders of man and animals. Adv Parasitol 3: 223-248.

50. Duffy MS, Miller CL, Kinsella JM, de Lahunta A (2004) *Parastrongylus cantonensis* in a nonhuman primate, Florida. Am J Trop Med Hyg 10: 2207-2210.

51. Teem JL, Qvarnstrom Y, Bishop HS, da Silva AJ, Carter J, et al. (2013) The occurrence of the rat lungworm, *Angiostrongylus cantonensis*, in nonindigenous snails in the Gulf of Mexico region of the United States. Hawaii J Med Public Health 72: 11-14.

52. Hochberg NS, Park SY, Blackburn BG, Sejvar JJ, Gaynor K, et al. (2007) Distribution of esosinophilic meningitis cases attributable to *Angiostrongylus cantonensis*, Hawaii. Emerg Infect Dis 13: 1675-1680.

53. Kim DY, Stewart TB, Bauer RW, Mitchell M (2002) *Parastrongylus* (=*Angiostrongylus*) *cantonensis* now endemic in Louisiana. J Parasitol 88: 1024-1026.

54. New D, Little MD, Cross J (1995) *Angiostrongylus cantonensis* infection from eating raw snails. N Engl J Med 332: 1105-1106.

55. Alicata JE, McCarthy DD (1964) On the incidence and distribution of the rat lungworm *Angiostrongylus cantonensis* in the Cook Islands, with observations made in New Zealand and Western Samoa. Can J Zool 42: 605-611.

56. Fontanilla IKC, Wade CM (2012) First report of *Angiostrongylus cantonensis* in the Giant African Land Snail *Achatina fulica* in French Polynesia detected using the SSU rRNA gene. Trop Biomed 29: 642-645.

57. Monte TCC, Simões RO, Oliveira AP, Novaes CF, Thiengo SC, et al. (2012) Phylogenetic relationship of the Brazilian isolates of the rat lungworm *Angiostrongylus cantonensis* (Nematoda: Metastrongylidae) employing mitochondrial COI gene sequence data. Parasit Vectors 5: 248-256.

58. Dorta-Contreras AJ, Padilla-Docal B, Moreira JM, Robles LM, Aroca JM, et la. (2011) Neuroimmunological findings of *Angiostrongylus cantonensis* meningitis in Ecuadorian patients. Arq Neuropsiquiatr 69: 466-469.
