# Supplementary material for: Global Decline in Suitable Habitat for Angiostrongylus ( = Parastrongylus) cantonensis: The Role of Climate Change
Source: PLoS One. 2014 Aug 14;9(8):e103831. doi: 10.1371/journal.pone.0103831 (PMC4133392; doi:10.1371/journal.pone.0103831)
Supplement: Table S2 — Bioclimatic variables used in the construction of the niche models. (DOCX) [file pone.0103831.s004.docx]

**Table S2.** **Bioclimatic variables used in the construction of the niche models.**

| **Variable** | **Definition** |
| --- | --- |
| BIO 1 | Annual mean temperature |
| BIO 2 | Mean diurnal range (Mean of monthly [max temp - min temp]) |
| BIO 3 | Isothermality (BIO 2 / BIO 7) * 100 |
| BIO 4 | Temperature seasonality (standard deviation * 100) |
| BIO 5 | Max temperature of warmest month |
| BIO 6 | Min temperature of coldest month |
| BIO 7 | Temperature annual range (BIO 5 - BIO 6) |
| BIO 8 | Mean temperature of wettest quarter |
| BIO 9 | Mean temperature of driest quarter |
| BIO 10 | Mean temperature of warmest quarter |
| BIO 11 | Mean temperature of coldest quarter |
| BIO 12 | Annual precipitation |
| BIO 13 | Precipitation of wettest month |
| BIO 14 | Precipitation of driest month |
| BIO 15 | Precipitation seasonality (coefficient of variation) |
| BIO 16 | Precipitation of wettest quarter |
| BIO 17 | Precipitation of driest quarter |
| BIO 18 | Precipitation of warmest quarter |
| BIO 19 | Precipitation of coldest quarter |
| Elevation | Elevation above sea level |
